# Berberine: A Promising Treatment for Neurodegenerative Diseases

**DOI:** 10.3389/fphar.2022.845591

**Published:** 2022-05-20

**Authors:** Ziqian Cheng, Chenglan Kang, Songtian Che, Jingyun Su, Qihan Sun, Tongtong Ge, Yi Guo, Jiayin Lv, Zhihui Sun, Wei Yang, Bingjin Li, Xin Li, Ranji Cui

**Affiliations:** ^1^ Jilin Provincial Key Laboratory on Molecular and Chemical Genetic, Second Hospital of Jilin University, Changchun, China; ^2^ Department of Cardiology, The China-Japan Union Hospital of Jilin University, Changchun, China; ^3^ Department of Orthopedics, The China-Japan Union Hospital of Jilin University, Changchun, China; ^4^ Department of Pharmacy, The First Hospital of Jilin University, Changchun, China

**Keywords:** berberine, neurodegenerative diseases, neuroinflammation, neuroprotection, oxidative stress

## Abstract

Berberine, as a natural alkaloid compound, is characterized by a diversity of pharmacological effects. In recent years, many researches focused on the role of berberine in central nervous system diseases. Among them, the effect of berberine on neurodegenerative diseases has received widespread attention, for example Alzheimer’s disease, Parkinson’s disease, Huntington’s disease, and so on. Recent evidence suggests that berberine inhibits the production of neuroinflammation, oxidative, and endoplasmic reticulum stress. These effects can further reduce neuron damage and apoptosis. Although the current research has made some progress, its specific mechanism still needs to be further explored. This review provides an overview of berberine in neurodegenerative diseases and its related mechanisms, and also provides new ideas for future research on berberine.

## 1 Introduction

Berberine (C_20_H_18_NO_4_, IUPAC name: 16,17-dimethoxy-5,7-dioxa-13-azoniapentacyclo [11.8.0.0^2,10^.0^4,8^.0^15,20^] henicosa-1(13),2,4(8),9,14,16,18,20-octaene, PubChem CID: 2353), an isoquinoline quaternary alkaloid, isolated from different medicinal plants with a molar weight of 336.36 g/mol, including *Hydrastis canadensis*, *Xanthorhiza simplicissima*, *Berberis aristata*, *Coptis chinensis*, *Coptis japonica*, etc. (Ortiz et al., 2014; Cicero and Baggioni, 2016; Feng et al., 2019). Berberine is a yellow powder, slightly soluble in ethanol or methanol (Kong et al., 2004; Wang et al., 2017). It has been reported that berberine is widely used in many traditional medical systems, including Ayurvedic, Iranian, and Chinese medicine (Kunwar et al., 2006; Cicero and Baggioni, 2016), and it has been used in some cases like cancer, diabetes, cardiovascular diseases, hypertension, Alzheimer’s disease, etc. (Imenshahidi and Hosseinzadeh, 2016; Wang et al., 2017; de Oliveira et al., 2019). Many preclinical studies conclusively shown that berberine plays a therapeutic role in many central nervous system disorders such as Alzheimer s disease, cerebral ischemia, depression, schizophrenia, epilepsy, and anxiety (Fan et al., 2017; Liu et al., 2017; Sedaghat et al., 2017; Fan J. et al., 2019a; Yuan et al., 2019; Rezaeian et al., 2020; Qiu et al., 2021; Zhao et al., 2021), however these experimental data are only obtained in animal models (Kulkarni and Dhir, 2010). Study elucidated that berberine can reduce the cognitive impairment caused by doxorubicin (DOX). In further research, it was found that berberine achieved antioxidant effects by reducing the expression of pro-inflammatory factors, apoptotic factors and nuclear transcription factor κ B (NF-κb), as well as up-regulating the expression of peroxlsome proliferator-activated receptor-γ coactlvator-1α (PGC-1α) and manganese superoxide dismutase. Berberine can also regulate cAMP response element binding protein (CREB) and brain-derived neurotrophic factor (BDNF) to further regulate synaptic plasticity (Shaker et al., 2021). Moreover, the literature points out that berberine has inhibitory effects on four key enzymes related to the pathogenesis of Alzheimer s disease: monoamine oxidase B (MAO-B), acetylcholinesterase, monoamine oxidase A (MAO-A), and butyrylcholinesterase (Yuan et al., 2019). However, the effects of berberine on neurodegenerative diseases are rarely reported and further investigation is still needed.

Due to the increase of the senior population in recent years, age-related diseases such as neurodegenerative diseases have become more common and pose a serious threat to human health (Heemels, 2016). Among them, World Health Organization reported that Alzheimer’s and other forms of dementia are currently among the top ten causes of death globally, ranking third in both the Americas and Europe in 2019 (World Health Organization, 2020). Moreover, neurodegenerative diseases, such as Alzheimer’s disease, Parkinson’s disease, bring a heavy burden to patients and their families, and become a public health problem that needs to be solved urgently. Neurodegenerative diseases are characterized by progressive damage to functions of synapses, neurons, glial cells (Marchesi et al., 2021; Pathak et al., 2021). Research on genome instability, autophagy, protein aggregation, and inflammation are currently a hot spot for the pathogenesis of neurodegenerative diseases (Hou et al., 2019). Although significant progress has been made in the research on the pathogenesis of neurodegenerative diseases such as Alzheimer’s disease, Parkinson’s disease, amyotrophic lateral sclerosis and Huntington’s disease, and many drugs have found neuroprotective effects in cells and animal models, but there is still no drug that can produce clinical changes significantly in the pathogenesis. It is very urgent to develop a drug that can effectively treat neurodegenerative diseases with few side effects.

Therefore, we conducted a systematic review of research articles on the role of berberine in neurodegenerative diseases over the past decade. The main focus is on the two most common neurodegenerative diseases, Alzheimer’s disease and Parkinson’s disease, other neurodegenerative diseases are also involved in a small amount. This review was carried out by searching on the electronic databases (including PubMed, ScienceDirect, and Pubchem) for studies focusing on the molecular mechanism of berberine in the biological processes related to neurodegenerative diseases. Furthermore, this review provides a perspective for future research that may contribute to the development of new drugs for neurodegenerative disease treatments.

## 2 Pharmacological Effects of Berberine

### 2.1 Oxidative Stress

The effect of berberine on a variety of neurodegenerative diseases indicates that it may have a regulatory effect on the common pathways of these diseases. MAO-B inhibitors are believed to have a positive effect on patients with Alzheimer’s and Parkinson’s disease (Mangoni et al., 1991; Rabey et al., 2000; Jiang et al., 2015a). The dopamine metabolic pathway mediated by MAO-B can generate hydrogen peroxide (H_2_O_2_), and with the increase of MAO-B activity, excessive H_2_O_2_ leads to oxidative stress and neuronal damage (Jiang et al., 2015a). Therefore, the antioxidant effect of berberine has great effects on the treatment of neurodegenerative diseases.

The pathogenesis of neurodegenerative diseases has several common features, such as oxidative damage and mitochondrial dysfunction. Among them, oxidative stress refers to the excessive accumulation of reactive oxygen species (ROS) that leads to the imbalance of oxidants and antioxidants, oxidative stress has the ability to lead to mitochondrial dysfunction and lysosome dysfunction, and ultimately induces neurodegenerative disease (Caspersen et al., 2005; Poprac et al., 2017; Li Z. et al., 2020a). It has been reported that Berberine significantly reduces ROS production in the cytoplasm and mitochondria (Sun et al., 2017). This effect may be related to the activation of AMP-activated protein kinase (AMPK) and sirtuin1 (SIRT1)/forkhead box O1 (FOXO1) pathway (Li et al., 2018; Ma et al., 2018). In addition, in PC12 and N2a cells, low-concentration administration of berberine significantly reduced the amount of ROS generation, lipid peroxidation, and DNA fragmentation, and at the same time increased glutathione content and superoxide dismutase activity (Sadeghnia et al., 2017). As we all know, the body has both enzymatic (such as glutathione-S-transferase) and non-enzymatic (such as thiols and reduced glutathione) antioxidant mechanisms to combat oxidative damage. Once ROS overwhelms the antioxidant activity of the cell, oxidative stress occurs (Lee et al., 2012; de Oliveira et al., 2019). Berberine-induced increase in heme oxygenase-1 (HO-1, an antioxidant enzyme) mRNA and protein expression is positively correlated with concentration and continuous administration time. This effect can be antagonized by kinase-protein kinase B (AKT) inhibitors and phosphatidylinositol 3-kinase (PI3K) inhibitors (Chen et al., 2012). An *in-vitro* study found that berberine protected NSC34 cells from H_2_O_2_-induced cytotoxicity by reducing ROS production, restoring glutathione, and superoxide dismutase activity, and activating the production of antioxidant proteins nuclear factor erythroid 2-related factor-2 (Nrf2) and HO-1 (Hsu et al., 2012; Fan D. et al., 2019a).

**FIGURE 1:**
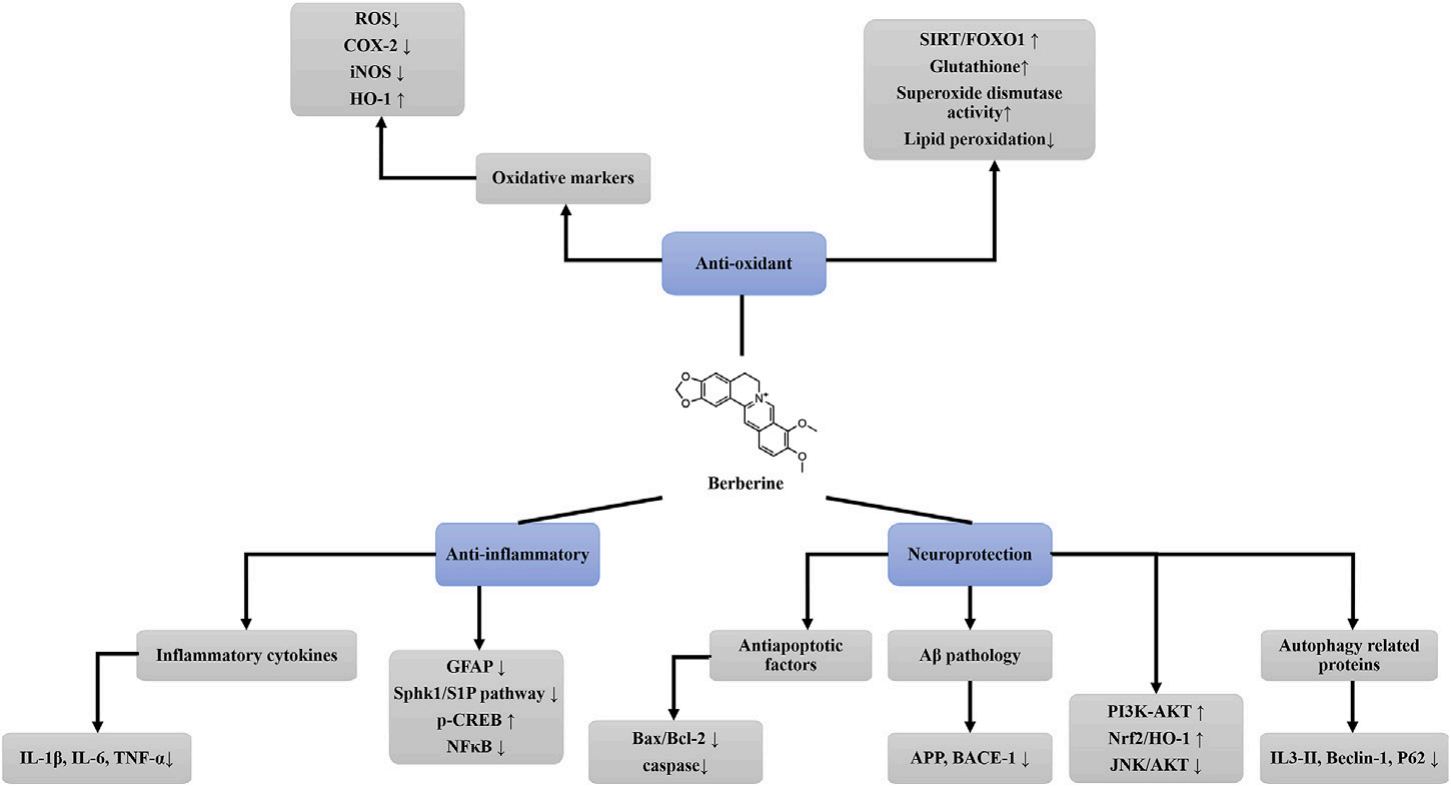
The pharmacological effects of berberine. This schematic drawing shows the anti-oxidant, anti-inflammatory and neuroprotective effects of berberine and its related molecular mechanisms. ROS, reactive oxygen species; COX-2, cyclooxygenase-2; iNOS, initric oxide synthase; HO-1, heme oxygenase-1; SIRT1, sirtuin1; FOXO1, forkhead box O1; IL-1β, interleukin 1β; IL-6, interleukin 6; TNF-α, tumor necrosis factor-alpha; GFAP, glial fibrillary acidic protein; SphK1, sphingosine kinase-1; S1P, sphingosine-1-phosphate; p-CREB, phosphorylated cAMP response element binding protein; NF-κb, nuclear transcription factor kappa B; Aβ, amyloid β; APP, amyloid precursor protein; BACE1, beta-secretase 1; PI3K, phosphatidylinositol 3-kinase; AKT, kinase-protein kinase B; Nrf2, nuclear factor erythroid 2-related factor-2; JNK, jun amino-terminal kinases.

Studies have found that berberine has an antidepressant effect (Kulkarni and Dhir, 2008; Fan et al., 2017), acute administration can increase dopamine, serotonin and norepinephrine levels in the whole brains of mice, and this effect can be antagonized by l-arginine or sildenafil pretreatment. In short, berberine can play an antidepressant effect by regulating brain biogenic amines and nitric oxide pathway (Kulkarni and Dhir, 2008). Besides, the treatment of berberine can reduce the activity of ecto-nucleoside triphosphate diphosphohydrolase (NTPDases) and 5′-nucleotidasein the cerebral cortex and hippocampus (de Oliveira et al., 2019). In brief, the application of berberine provides a promising neuroprotective therapy for neurodegenerative diseases induced by oxidative damage, such as Alzheimer’s disease, Parkinson’s disease (Figure 1). It may be playing an antioxidant role by reducing ROS, iNOS, COX-2, increasing HO-1, and activating SIRT/FOXO1 signal pathway. Berberine can also play a therapeutic role by increasing glutathione and superoxide dismutase activity. In addition, whether the antioxidant effect of berberine alters other markers needs to be further explored.

### 2.2 Neuroprotection

*In vitro* experimental results show that berberine can reduce 6-hydroxydopamine-induced reactive oxygen generation and caspase-3 activation in SH-SY5Y cell. In addition, berberine can induce activation of PI3K/AKT signaling pathway, which is related to Nrf2 expression and neuroprotection (Bae et al., 2013). Similarly, it has been confirmed in PC-12 cells that berberine exerts a neuroprotective effect by activating the PI3K/AKT signaling pathway and Nrf2/HO-1 antioxidant signaling pathway (Zhang et al., 2017). In BV2 and N2a cells treated with amyloid β (Aβ), berberine promotes cell proliferation, inhibits the activity of caspase-3, and reduces the rate of apoptosis, in addition, berberine promotes cell viability *via* the microRNA (miR)-188/nitric oxide synthase 1 (NOS1) pathway (Chen M. et al., 2020a). By analyzing the learning and memory of post-cerebral ischaemia. animals, the researchers discovered the neuroprotective effects of berberine (Ye et al., 2009; Zhang C. C. et al., 2018a). *In vitro* experiments (organotypic hippocampal culture exposed to oxygen and glucose deprivation), the culture treated with berberine has less cellular death. In further research, it was found that the neuroprotection mediated by berberine after ischemia involves the AKT/glycogen synthase kinase-3β (GSK3β)/extracellular signal-regulated kinase 1/2 (ERK 1/2) signal pathway, and inhibits Jun amino-terminal kinases (JNK) and caspase-3 activity (Simões Pires et al., 2014).

Berberine inhibits neuronal apoptosis by regulating autophagy-related proteins [Microtubule-associated protein 1A/1B-light chain 3 (LC3), Beclin-1, p62], and apoptosis regulatory proteins [caspase 3, caspase 8, caspase 9, poly ADP-ribose polymerase (PARP), and B-cell lymphoma 2 (Bcl-2)/Bcl-2 associated X (Bax)] (Zhang et al., 2016). Similarly, berberine shows anti-apoptotic effects by reducing the overexpression of caspase-3 and Bax/Bcl-2 (Sadeghnia et al., 2017). In 1-methyl-4-phenyl-1,2,3,6-tetrahydropyridine/probenecid (MPTP/P)-induced Parkinson’s disease model mice, MPTP/P injection increased the ratio of Bax/Bcl-2 and the expression of caspase-3 in the hippocampus, while berberine administration reversed these effects (Kim et al., 2014).

In addition, animal experiments and cell experiments have confirmed that berberine reduces the levels of extracellular and intracellular Aβ, increases the levels of LC3-II, Beclin-1, hVps34 and cathepsin-D, and reduced the levels of P62, Bcl-2, amyloid precursor protein (APP), and beta-secretase 1 (BACE1). Briefly, berberine plays a neuroprotective effect by promoting autophagy clearance and reducing Aβ production (Huang et al., 2017).

Besides, it is indicating that berberine facilitates nerve regeneration via JNK-AKT signal pathway mediated by insulin-like growth factor receptor (Zhang H. N. et al., 2018b). In astrocytes, berberine reduces glutamate-induced cytotoxicity by reducing mitochondrial fragmentation and neurodegeneration (Campisi et al., 2011). Additionally, berberine prolongs the incubation period of epileptic seizures in a dose-dependent manner. Long-term administration of berberine reduces the level of malondialdehyde in epileptic mice and enhances the activities of glutathione and catalase (Gao et al., 2014). In addition, the neuroprotective role of berberine is related to the decrease in endoplasmic reticulum stress and oxidative stress (Liang et al., 2021). Berberine can also be used in combination with other drugs such as levetiracetam and curcumin to enhance its neuroprotective effect by reducing inflammation and oxidative stress (Lin et al., 2020; Singh et al., 2021). *In vitro*, low-dose berberine significantly increased PC12 cell viability, whereas high-dose berberine did the opposite (Zhang et al., 2017). Additionally, low-dose berberine protected PC12 cells from 6-hydroxydopamine (6-OHDA, Parkinson’s disease-related neurotoxin)-induced cytotoxicity and apoptosis, whereas high-dose berberine showed no neuroprotective activity. Further experiments found that low-dose berberine can promote cell survival and antioxidant effects by up-regulating PI3K/AKT/Bcl-2 and Nrf2/HO-1 signal pathways. In animal studies, low-dose berberine attenuated 6-OHDA-induced loss of dopaminergic neurons and hypokinesia in zebrafish, whereas high-dose berberine had no apparent effect (Zhang et al., 2017). These suggests that the neuroprotective effects of berberine may be related to an excitatory mechanism that promotes cell survival and antioxidant-related signaling pathways. Studies have found that berberine attenuates ischemia-reperfusion injury through NF-κB nuclear translocation in mice with transient cerebral artery occlusion, however, this neuroprotective effect was more pronounced at the high dose (50 mg/kg) than the low dose (25 mg/kg) (Zhu et al., 2018). These differences may be related to different concentrations of berberine and different models of nerve injury. In addition, berberine *in vivo* may be metabolized to make its active ingredients different from those used *in vitro*, resulting in inconsistent results.

In brief, berberine can reduce anti-apoptotic factors (Bax/bcl-2, caspase), autophagy related proteins (IL-3, Beclin, etc.), Aβ pathology, and activate PI3K-AKT and other signaling pathways, and then produce neuroprotective effect (Figure 1). However, whether berberine alleviates neurodegenerative disease symptoms by increasing neuroprotection requires further study.

### 2.3 Neuroinflammation

Some studies have shown that berberine activates macrophages and increases their phagocytic function, increases the production of interleukin (IL)-1, and enhances non-specific immunity, can be used as a neuroprotective agent to prevent Alzheimer’s disease (Kumazawa et al., 1984; Panahi et al., 2013). Long-term administration of berberine increases the expression of IL-1β and inducible nitric oxide synthase (iNOS) in Alzheimer’s disease mice hippocampus, and ameliorate memory impairment (Zhu and Qian, 2006). Besides, berberine down-regulates the expression of acetylcholinesterase and inhibits acetylcholinesterase activity in Alzheimer’s disease model mice hippocampus which induced by heavy metals, in addition, berberine normalizes the production of inflammatory factors, such as tumor necrosis factor-alpha (TNF-α), IL-6, IL-1β (Hussien et al., 2018).

Studies demonstrated that berberine contributed to AMPK signaling pathway activation and participate in anti-neuroinflammation. In BV-2 microglia, berberine down-regulates lipopolysaccharide- or interferon-γ-induced iNOS and cyclooxygenase-2 (COX-2) level, and inhibits inflammatory cytokines expression (for example IL-6, IL-1β, TNF-α) (Lu et al., 2010). On the other hand, berberine can inhibit ERK phosphorylation (Lu et al., 2010), and induce the phosphorylation of Liver kinase B1 (LKB1) (Ser428), calcium/calmodulin-dependent protein kinase II (CaMKII) (Thr286), and AMPK (Thr172) (Lu et al., 2010). In animal experiments, berberine has the ability to improve learning and memory impairment in rats via lipopolysaccharide. Further studies showed that berberine enhanced the activities of glutathione peroxidase, glutathione, superoxide dismutase, and catalase, further reducing the activity of acetylcholinesterase and caspase-3, protein carbonyl, and DNA fragmentation in the hippocampus (Sadraie et al., 2019). In addition, berberine can properly restore the levels of 3-nitrotyrosine (3-NT), COX 2, glial fibrillary acidic protein (GFAP), and sirtuin 1 in the hippocampus (Sadraie et al., 2019). Besides, the protective effect of berberine resisting MTPT-induced toxicity may be related to the enhancement of autophagy by berberine through AMPK-dependent pathways (Deng and Ma, 2021).

The study found that berberine alleviated the severity of symptoms in the multiple sclerosis mouse model, and in primary astrocyte culture, berberine inhibited the increase in sphingosine kinase-1 (SphK1), and Sphingosine-1-phosphate (S1P) induced by lipopolysaccharide. A number of researchers have reported that the activation of the SphK1/S1P signaling pathway marks the occurrence of autoimmune diseases, and the up-regulation of SphK1 is related to the pathogenesis of multiple sclerosis (Luo et al., 2017). In Alzheimer’s disease mice, GFAP and neuron loss increased and neuronal nuclei (NeuN) decreased significantly. Studies have found that under pathological conditions, astrocytes are activated by Aβ to produce cytoinflammatory factors, complement, oxygen free radicals, etc., which then trigger inflammation, promote nerve cell damage and death, and ultimately aggravate Alzheimer’s disease (Do et al., 2014; Matuszyk et al., 2021). The latest research shows that berberine significantly decreases the expression of GFAP in the Alzheimer’s disease mice hippocampus, indicating berberine inhibits the overexpression of astrocytes in Alzheimer’s disease mice. These may be linked to the effect of berberine on improving local blood flow, reducing the production of Aβ and reducing cell apoptosis (Ye et al., 2021). Similarly, berberine treatment can significantly reduce the oxidative stress in diabetic rat hippocampus and suppress GFAP immunoreactive astrocytes (Moghaddam et al., 2014). Astrocytes treated with berberine can increase the expression of p85 and p-AKT. Berberine also increases the accumulation of Nrf2 and DNA binding activity in the nucleus. It is worth noting that the increase in Nrf2 DNA binding activity induced by berberine can be antagonized by PI3K inhibitors and AKT inhibitors. In other words, in astrocytes. the increase in HO-1 expression induced by berberine is activated by Nrf2 activation through PI3K/AKT signaling pathway (Chen et al., 2012). Similarly, in SH-SY5Y cells, berberine resists rotenone-induced neurotoxicity through antioxidant effects and activates the PI3K/AKT signaling pathway (Deng et al., 2020).

Studies have found that the combined treatment of DOX and berberine can significantly counteract the increase in acetylcholinesterase activity, oxidative stress, the decrease in glutathione content and CAT activity, and the increase in GFAP, NF-κB and caspase-3 induced by DOX (Ibrahim Fouad and Ahmed, 2021). Zhang et al. found that berberine can increase the levels of neuroprotective factors, such as p-AKT and p-CREB, and down-regulate inflammatory response factors to inhibit inflammation, such as NF-κB (Zhang et al., 2012).

Neuroinflammation plays a pivotal role in the pathogenesis of neurodegenerative diseases. It is a central feature in the neurodegenerative process that leads to more neuronal loss over time. In previous studies, it was found that berberine can reduce the expression of inflammatory factors and GFAP, while inhibiting the Sphk1/S1P signaling pathway and activating CREB signaling pathway. These show that berberine is very likely to play a therapeutic role in neurodegenerative diseases by inhibiting neuroinflammation. However, there are few literatures about the effect of berberine on astrocytes, and further experimental verification is needed.

## 3 Therapeutic Effects of Berberine on Neurodegenerative Disease

In recent years, many studies surrounded the effect of berberine in central nervous system disease, including Alzheimer’s disease, Parkinson’s disease, Huntington disease, etc. (Jiang et al., 2011; Kim et al., 2014; Jiang et al., 2015b; Bandiwadekar et al., 2021). Berberine has proven its protective role on oxidative stress, neuroinflammation, neuroprotection, etc. (Zhang et al., 2012; Maleki et al., 2018; de Oliveira et al., 2019; Li Z. et al., 2020a; Qin et al., 2020; Rezaeian et al., 2020; Mohi-Ud-Din et al., 2021). And whether it can be used to improve the symptoms of neurodegenerative diseases has attracted widespread attention.

### 3.1 Alzheimer’s Disease

Alzheimer’s disease, an age-related neurodegenerative disease, affects the quality of life of patients, and their families. Patient with Alzheimer’s disease suffer from memory loss, cognitive decline, energy metabolism dysregulation, changes in personality, and behavior (Goedert and Spillantini, 2006; Ribeiro et al., 2017; Shinjyo and Kita, 2021). Although a considerable new research progress has been made in Alzheimer’s disease research, the pathogenesis remains unclear. Current typical histopathological changes in Alzheimer’s disease include Aβ plaques and tau tangles, the accumulation of these proteins leads to neuroinflammation and normal dysfunction, leading to neuronal death (Ameen and Michniak-Kohn, 2017; Bandiwadekar et al., 2021; Ye et al., 2021). The hypotheses on the pathogenesis of Alzheimer’s disease include cholinergic hypothesis, amyloid toxicity hypothesis, oxidative stress hypothesis, etc. (Ye et al., 2009; Guo et al., 2021). At present, the therapies of Alzheimer’s disease include stem cell therapy, gene therapy, and chemotherapy, but these therapies have limitations inevitably such as inefficient and neurotoxicity (Bandiwadekar et al., 2021). At the same time, natural compounds have better compatibility with the human body and have fewer side effects. A number of studies have shown that some natural drugs have significant neuroprotective, anti-oxidant and anti-inflammatory characteristics, and are suitable for the treatment of different types of neurodegenerative diseases (Ahmed et al., 2015; Jiang et al., 2015b; Fan et al., 2017; Zhang et al., 2020). Many literatures have reported the improvement effect of berberine on Alzheimer’s disease (Chen M. et al., 2020a; Chen Y. et al., 2020b; Xuan et al., 2020; Raju et al., 2021).

Aβ has a neurotoxic effect, which can lead to neuronal degeneration, death, apoptosis, and decrease in the number of synapses and ultimately lead to cognitive impairment and behavioral abnormalities (Li W. et al., 2020b; Ye et al., 2021). It has proved that activating microglia by reducing Aβ is an effective treatment for Alzheimer’s disease. Microglia exerts a vital role on the repair of central nervous system damage. In Alzheimer’s disease, the production of pro-inflammatory factors and Aβ can over-activate microglia, secrete inflammatory factors and neurotoxins, and then induce neuronal damage and even apoptosis, thereby triggering Alzheimer’s disease. On the contrary, microglia can also protect the central nervous system by engulfing Aβ, slowing the development of Alzheimer’s disease (Li et al., 2021). Study have shown berberine can inhibit Aβ-induced microglial activation mediated by suppressor of cytokine signaling 1 (SOCS1) (Guo et al., 2021). Berberine induces the production of antioxidant Aβ40 and inhibits the formation of Aβ42, which is the cause of Aβ plaque accumulation, and then exerts a neuroprotective effect (Hussien et al., 2018). Besides, berberine inhibits the p-ERK/Eukaryotic translation initiation factor 2alpha (eIF2α) pathway and then reduces BACE1 expression, the decrease in BACE1 protein expression inhibits the production of Aβ. In other words, berberine suppressed Aβ production via inhibiting protein kinase R (PKR)-like endoplasmic reticulum kinase (PERK)/eukaryotic translation-initiation factor 2α (elF2α) signaling-mediated BACE1 translation (Liang et al., 2021). Berberine also restored protein phosphate 2A (PP2A) activity and glycogen synthase kinase 3 (GSK-3β) activity (GSK-3β Tyr216 and Ser9 site phosphorylation increased) (Yu et al., 2011).

**FIGURE 2:**
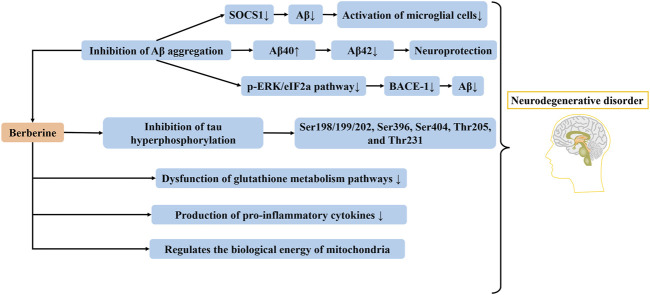
The therapeutic effect of berberine on Alzheimer’s disease. Berberine inhibits Aβ aggregation, tau hyperphosphorylation and pro-inflammatory cytokines production, in addition to regulating glutathione metabolism pathways and mitochondrial bioenergetics. SOCS1, suppressor of cytokine signaling 1; Aβ, amyloid β; BACE1, beta-secretase 1; p-ERK, phosphorylated extracellular signal-regulated kinase.

Studies have shown that the severity of Alzheimer’s disease has a connection with the degree of neurofibrillary tangles deposition, and most neurofibrillary tangles are caused by abnormal hyperphosphorylation of tau (Šimić et al., 2016; Šimić et al., 2017; Chen Y. et al., 2020b). In the meantime, a number of researchers have reported that tau dysfunction can trigger the production of Aβ, and the overproduction of Aβ and tau dysfunction synergistically induce Alzheimer’s disease (Rajmohan and Reddy, 2017). Berberine can alleviate cognitive function of Alzheimer’s disease mice via reducing the hyperphosphorylation of tau and promoting the autophagy clearance of tau (Chen Y. et al., 2020b). After 24 h of berberine administration, the hyperphosphorylation of tau at Ser198/199/202, Ser396, Ser404, Thr205, and Thr231 was significantly reduced (Yu et al., 2011). Besides, the effects of berberine in Alzheimer’s mice may be related to the inhibition of NF-κB activity and lipid peroxidation, and the increase of glutathione activity in the hippocampus (He et al., 2017) (Figure 2).

*In vitro*, berberine can regulate mitochondrial bioenergetics, reduce primary energy and glutathione metabolic pathway dysfunction, inhibit basal respiration, and suppress the production of pro-inflammatory cytokines. Moreover, berberine and pioglitazone have similar binding affinity to peroxisome proliferator-activated receptor gamma (PPARγ) protein, have an overlapping effect on Alzheimer’s disease (Wong et al., 2021).

Under pathological conditions, long-term excessive stress can cause damage to the endoplasmic reticulum function, and endoplasmic reticulum stress activates the apoptosis pathway, which leads to nerve cell damage and even death (Cameron, 2013). Berberine treatment enhanced the cognitive ability in Alzheimer’s disease rats, this effect is related to the reduction of endoplasmic reticulum stress. Berberine regulates the mRNA levels of glucose-regulated protein 78 (GRP78), CCAAT/enhancer binding protein (C/EBP) -homologous protein (CHOP), procaspase-3/9/12 in Alzheimer’s disease rat’s hippocampus, which involve in the endoplasmic reticulum stress pathway (Xuan et al., 2020).

Berberine conjugates, such as berberine-benzenediol derivatives, berberine-melatonin hybrid, and berberine-ferulic acid hybrid, exhibited anti-oxidant activity, and inhibited Aβ aggregation. Among them, the hydroquinone-berberine hybrid, had the greatest ability to suppress Aβ aggregation and inhibit oxidative stress (Jiang et al., 2011).

### 3.2 Parkinson’s Disease

Parkinson’s disease, a neurodegenerative disease characterized by muscle stiffness, tremor, speech and gait changes, and its risk factors include environmental factors, genetic factors, gender factors, age, etc. (Kalia and Lang, 2015; Hou et al., 2019). At present, the therapy of Parkinson’s disease mainly uses dopamine agonists and MAO-B inhibitors to suppress the decomposition of dopamine, but they only target symptoms and have serious side effects (Jiang et al., 2015a). In the transcriptomics of *Microsporum canis*, there are 6 signaling pathways that have significant changes in Gene Ontology functional enrichment analysis after berberine treatment, including steroid biosynthesis, steroid hormone biosynthesis, Parkinson’s disease, 2,4-Dichlorobenzoic acid (2,4-DCBA) degradation and biosynthesis of tropane, piperidine and isoquinoline alkaloids (Xiao et al., 2015).

Previous studies have found that berberine inhibits cell death induced by 6-OHDA, and increases the expression of HO-1, ultimately protecting dopaminergic neurons (Bae et al., 2013). In SH-SY5Y cells, berberine suppressed 6-OHDA-induced ROS production, caspase-3 activation, and cell death (Bae et al., 2013). These indicate that berberine can be used as an effective therapeutic agent for dopaminergic neuron degeneration. In MPTP/P-induced mouse model of Parkinson’s disease, berberine reduced neuron loss in the substantia nigra pars compacta, dopaminergic fiber loss in the striatum, and apoptosis in the hippocampus. Animal behavior experiments have shown that the disorder of movement balance and coordination has been improved (Kim et al., 2014). However, when berberine is long-term administered to 6-OHDA-induced rat model of Parkinson’s disease, monitoring for adverse symptoms is required. Preclinical studies have found that berberine increases the number of tyrosine hydroxylase (TH)-positive neurons in the substantia nigra, at the same time, berberine also increases striatum dopamine, norepinephrine, 3,4-dihydroxyphenylacetic acid (DOPAC) and homovanillic acid levels (Shin et al., 2013). Berberine is a potential therapeutic drug for alleviating motor dysfunction and memory impairment in patients with Parkinson’s disease (Kim et al., 2014). Meanwhile, there are several studies offer some important insights into the neurotoxic effects of berberine. It is reported that in the Parkinson’s disease model rats induced by 6-OHDA, berberine aggravate the degeneration of dopaminergic neuron in the substantia nigra of rats (Shin et al., 2013). In addition, berberine can aggravate the cytotoxicity induced by 6-OHDA in PC12 cells and aggravate of dopaminergic neuron death (Kwon et al., 2010).

Interestingly, Wang et al. pointed out that the application of berberine in Parkinson’s disease may up-regulate the synthesis of L-dopa in the intestinal microbiota through vitamin-like effects, thereby exerting a therapeutic effect (Wang Y. et al., 2021a). Besides, berberine can prevent NACHT, LRR, and PYD domains-containing protein 3 (NLRP3) inflammasome from being activated during the inflammation process of Parkinson’s disease and restore autophagy activity to protect dopamine neurons (Huang et al., 2020).

In addition to the above studies, in the zebrafish Parkinson’s disease model, berberine derivatives can be used to treat Parkinson’s disease. This is because berberine derivatives can cross the blood-brain barrier and target mitochondria in the meantime (Wang L. et al., 2021b).

### 3.4 Other Neurodegenerative Diseases

Huntington’s disease is mainly caused by an autosomal dominant mutation in either of the two copies of the Huntingtin (HTT) gene (Cicchetti et al., 2014; Fan D. et al., 2019b). The pathogenesis of Huntington’s disease is mainly related to striatal atrophy and neuron loss. Its symptoms include chorea, dystonia, cognitive decline, etc. (Duff et al., 2007; Subramaniam et al., 2009; Hou et al., 2019). In previous studies, the expansion of polyglutamine (polyQ) bundles to 36 or more glutamine repeats will cause the HTT protein to misfold and aggregate, leading to neuronal death and symptoms of Huntington’s disease (Mangiarini et al., 1996). Recent evidence suggests that the autophagy-lysosome pathway is associated with the removal of aggregated polyQ-HTT (Sarkar and Rubinsztein, 2008; Fan D. et al., 2019b). Studies have found that berberine can effectively alleviate the motor dysfunction of HTT mice and prolong their survival time, further studies proves that berberine promote the degradation of mutant HTT protein through enhancing autophagy function, thereby reducing the accumulation of mutant huntingtin protein (Jiang et al., 2015b). Moreover, recent advances have revealed that berberine and its derivatives promote the elimination of neurotoxic misfolded proteins, which can be used as a potential treatment for neurodegenerative diseases (Rusmini et al., 2020).

Multiple sclerosis, characterized by multiple demyelinating lesions of the spinal cord and brain, has a progressive neurodegenerative pattern (Manu et al., 2021; Zahid et al., 2021). Berberine has been reported as a potential drug for the treatment of multiple sclerosis. In experimental autoimmune encephalomyelitis (a model of multiple sclerosis), berberine reduces the permeability of the blood-brain barrier and inhibits the activity of matrix metalloprotease 9 (MMP-9) in the cerebrospinal fluid and the brain of model mice (Ma et al., 2010). At the same time, berberine can inhibit gelatinase activity and reduce laminin degradation (Jiang et al., 2013). In addition, the severity of multiple sclerosis is positively correlated with the severity of reactive gliosis, and whether the anti-inflammatory effect of berberine has a connection with the treatment of multiple sclerosis still remains to be explored (Luo et al., 2017).

## 4 Limitations of Berberine Treatment

Berberine has a variety of activities that may be involved in alleviating Alzheimer’s disease, including antioxidant, neuroprotective, and anti-inflammatory effects (Campisi et al., 2014). As a natural pharmaceutical ingredient, berberine has the advantage of high safety (Rabbani et al., 1987), it is generally considered to be non-genotoxic and cytotoxic in clinical (Imanshahidi and Hosseinzadeh, 2008). So far, there have been no reports of serious adverse reactions caused by oral berberine in clinical practice, and in short-term and long-term trials, berberine is safe for most human subjects (Liu et al., 2016; Wang et al., 2017). In addition, berberine is small in size, so it can available through the blood-brain barrier and effectually act on molecular target, showing great therapeutic potential in the treatment of neurodegenerative diseases (Wang et al., 2005; Ji and Shen, 2011; Jiang et al., 2015a). However, due to the disadvantages of poor water solubility and solubility, the bioavailability of oral berberine is low (Kumar et al., 2015). At present, studies have designed formulations aimed at improving its bioavailability. For example, oral microemulsions containing berberine are 6.47 times more bioavailable than tablets and suspensions (Gui et al., 2008). Furthermore, studies have found that verapamil can enhance the neuroprotective effect of berberine by inhibiting the mechanism of P-glycoprotein efflux and preventing changes in calcium homeostasis (Kumar et al., 2016). Therefore, the changes in the dosage form or the combination with other drugs can further compensate for the weakness of low bioavailability of berberine.

## 5 Conclusion

The above evidences suggest that berberine has various roles in neurological diseases, including neuroinflammation, neuroprotection, oxidative stress, etc. At present, few studies to date have reported the pharmacological effects of berberine on neurodegenerative diseases, especially in human trials. In addition, most of the studies on berberine’s use in the treatment of neurodegenerative diseases focus on Alzheimer’s disease and Parkinson’s disease, and few other diseases are involved. Therefore, the answers to some questions remain unclear. Berberine may be a valuable potential therapeutic target for various neurodegenerative diseases, as data based on both *in vitro* models and animal models of neurodegenerative diseases support the beneficial effects of berberine. In addition, it is easy to introduce natural products containing berberine into the diet because they are common enough to be used preventively. However, further research is still needed to fully understand the efficacy and dosage of berberine in clinical trials.
